# Supplementary material for: Recombinant Oil-Body-Expressed Oleosin-hFGF5 in Arabidopsis thaliana Regulates Hair Growth
Source: Genes (Basel). 2022 Dec 22;14(1):21. doi: 10.3390/genes14010021 (PMC9858518; doi:10.3390/genes14010021)
Supplement: Supplementary file 1 [file genes-14-00021-s001.zip › genes-2018509-supplementary.pdf]

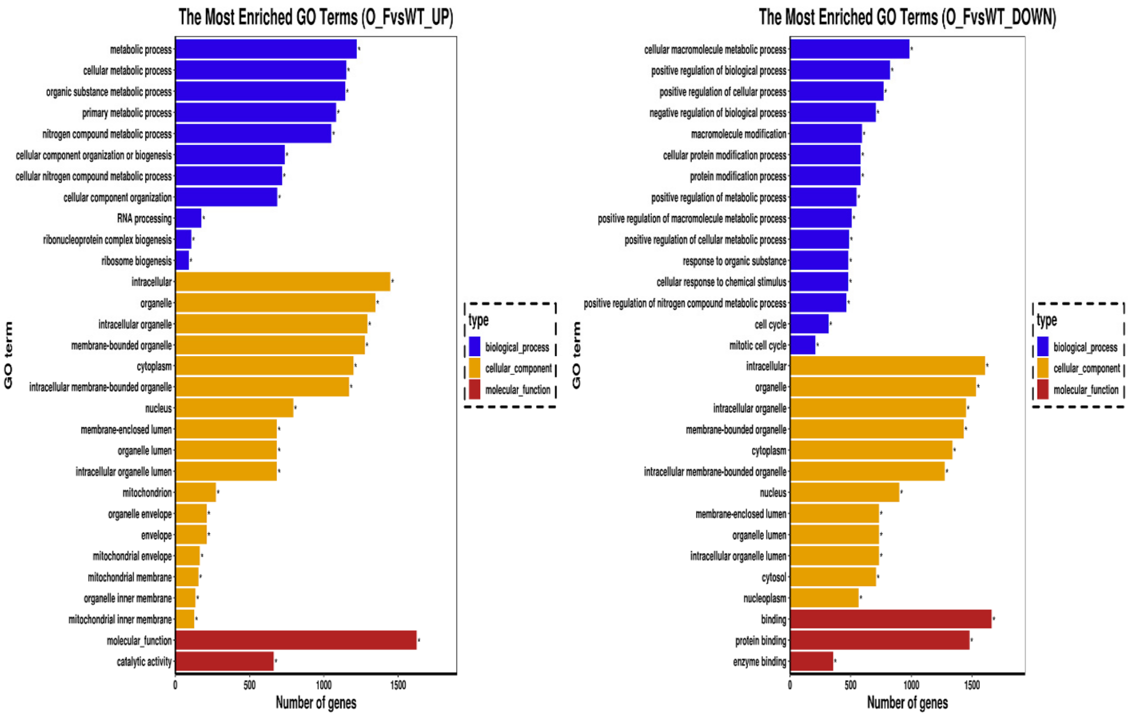

Figure S1. Differential gene enrichment analysis by KEGG.

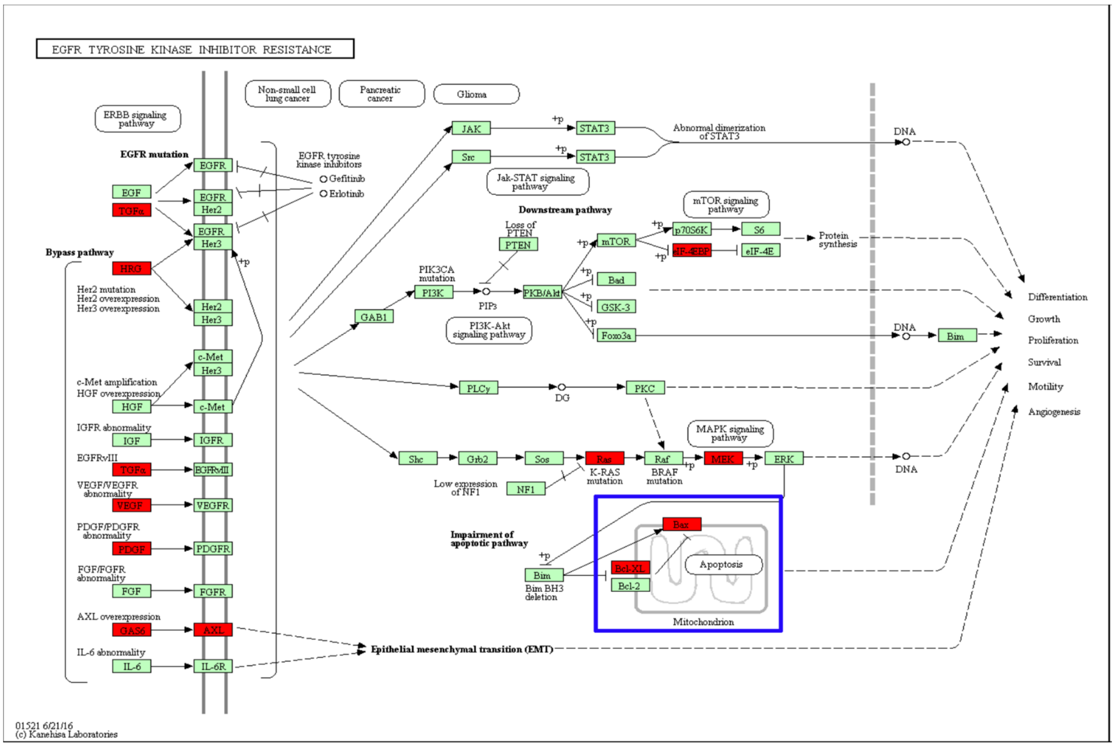

Figure S2. The recombinant oil body expressing hFGF5 regulated Bax and participates in EGFR pathway. The blue box indicates that the target gene is involved in the regulation process, and the red marked gene indicates that the up-regulation of gene expression induced by ROBF5 treatment may be involved in the EGFR pathway.

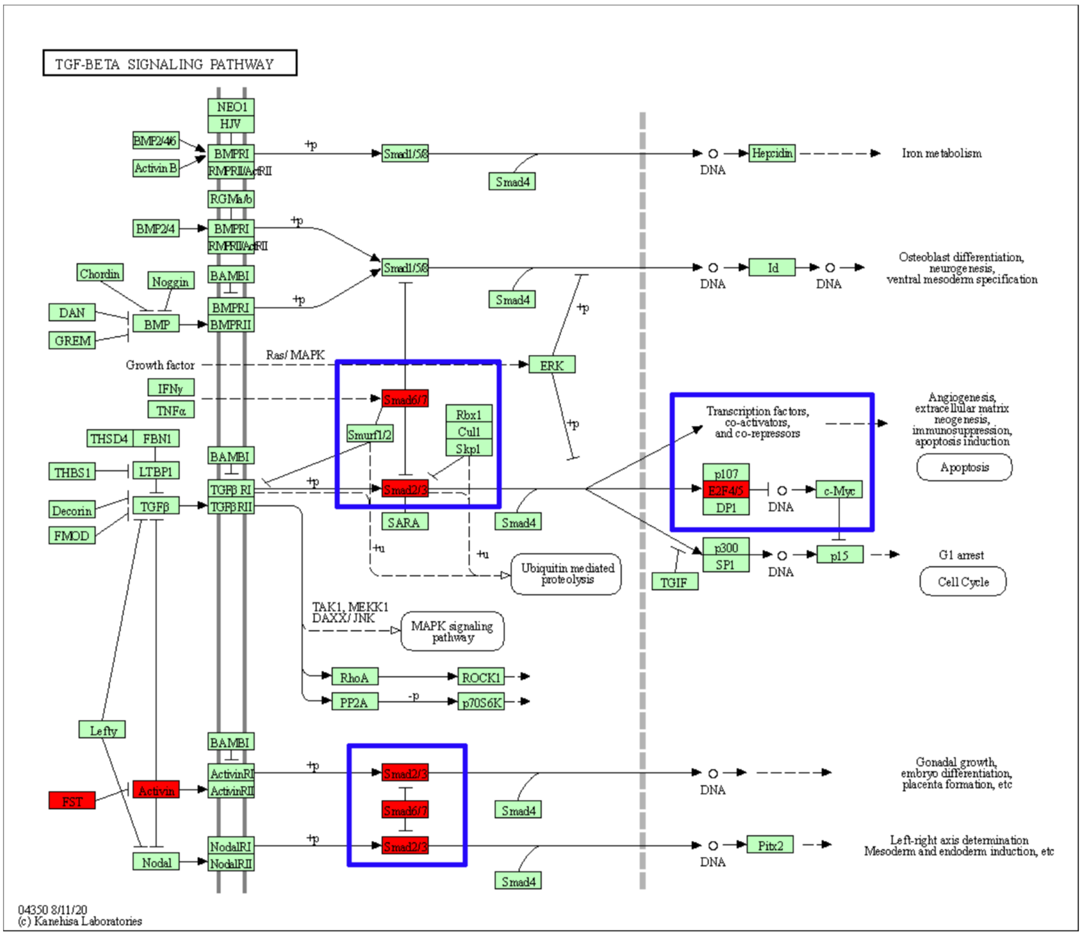

Figure S3. The recombinant oil body expressing hFGF5 regulates E2F4, E2F5, Smad7 and participates in TGF-β pathway. The blue box indicates that the target gene is involved in the regulation process, and the red marked gene indicates that the up-regulation of gene expression induced by ROBF5 treatment may be involved in the TGF-β pathway.

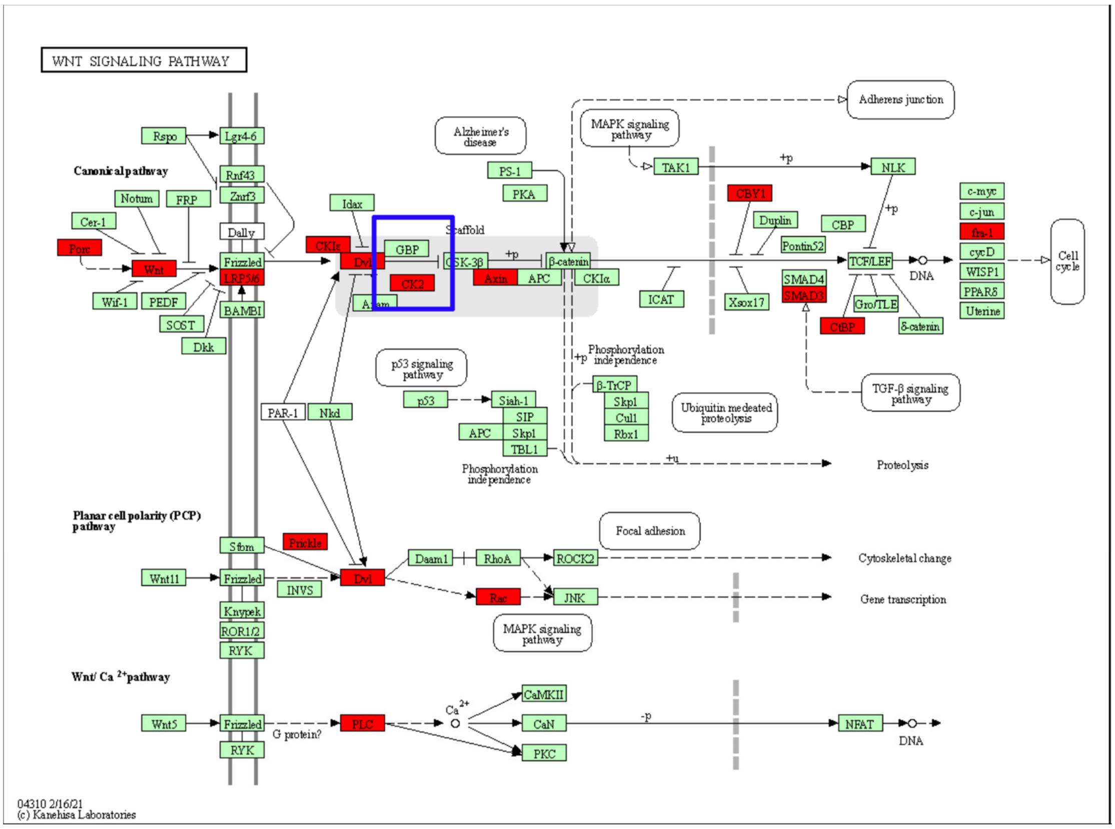

Figure S4. The recombinant oil body expressing hFGF5 regulates CSNK1 and participates in Wnt pathway. The blue box indicates that the target gene is involved in the regulation process, and the red marked gene indicates that the up-regulation of gene expression induced by ROBF5 treatment may be involved in the Wnt pathway.

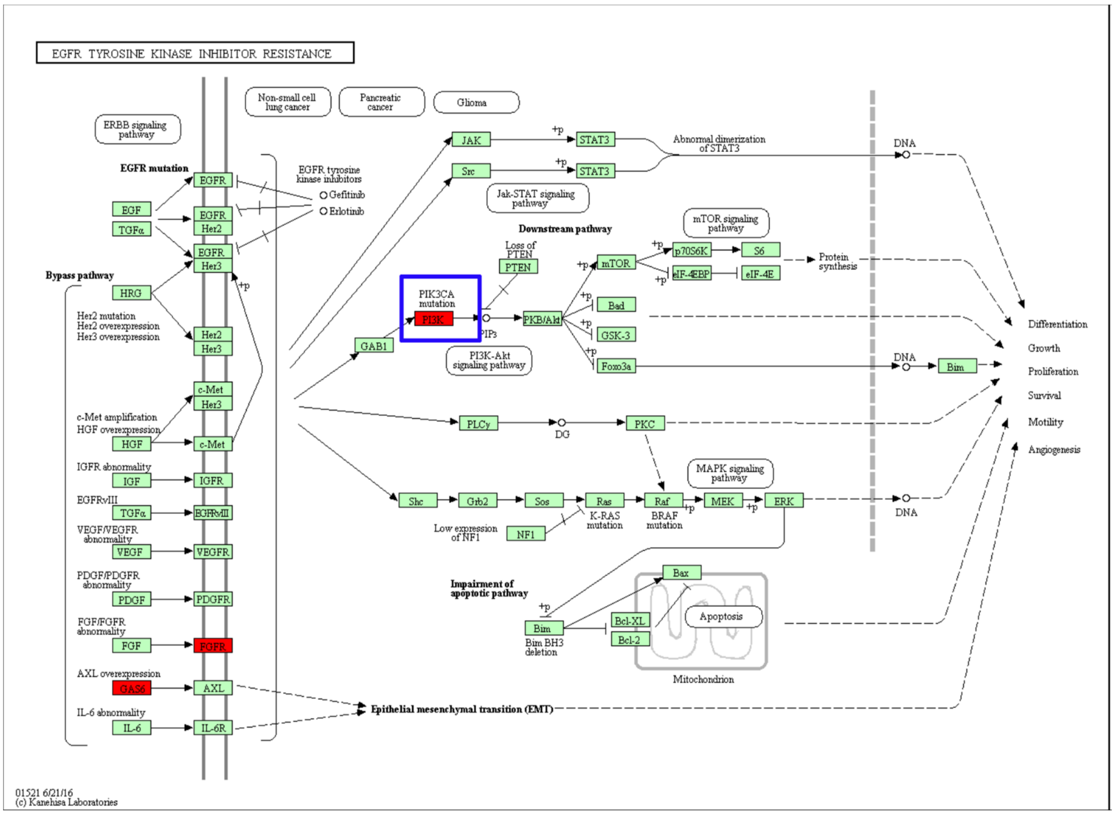

Figure S5. The recombinant oil body expressing hFGF5 regulates PIK3R3 and participates in EGFR pathway. The blue box indicates that the target gene is involved in the regulation process, and the red marked gene indicates that the down-regulation of gene expression induced by ROBF5 treatment may be involved in the EGFR pathway.

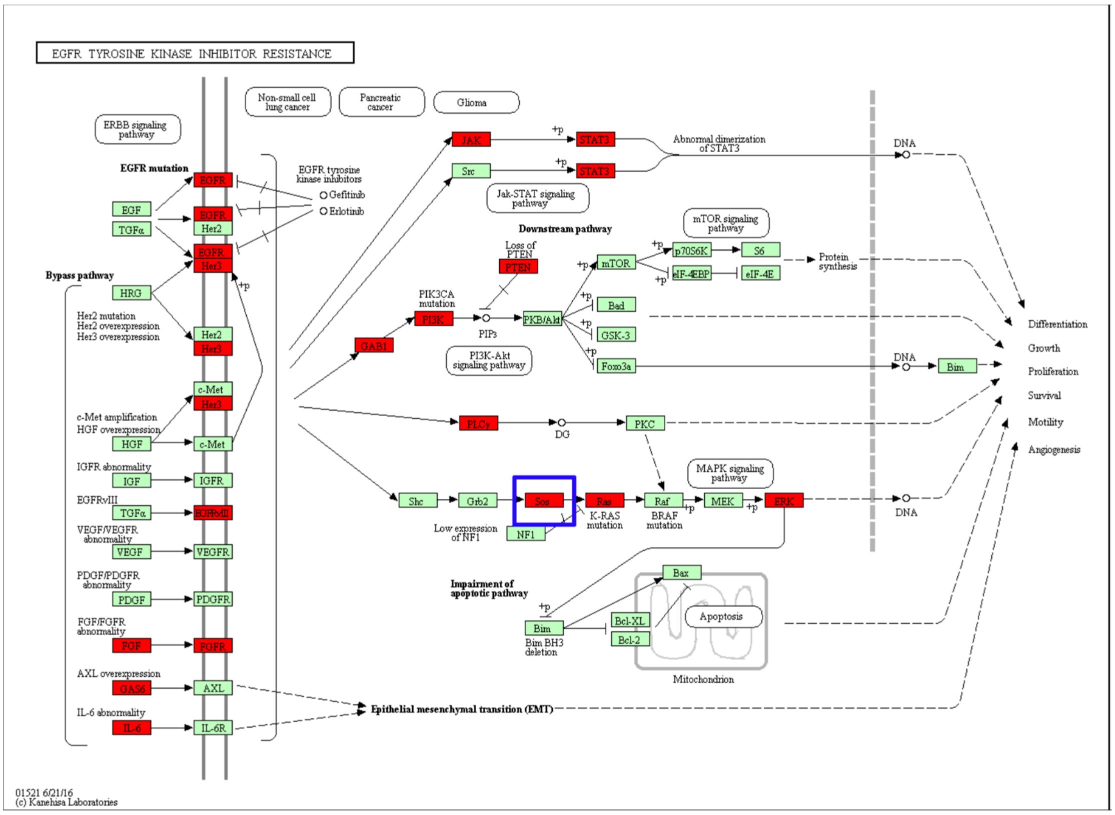

Figure S6. The recombinant oil body expressing hFGF5 regulates SOS1 and participates in EGFR pathway. The blue box indicates that the target gene is involved in the regulation process, and the red marked gene indicates that the down-regulation of gene expression induced by ROBF5 treatment may be involved in the EGFR pathway.

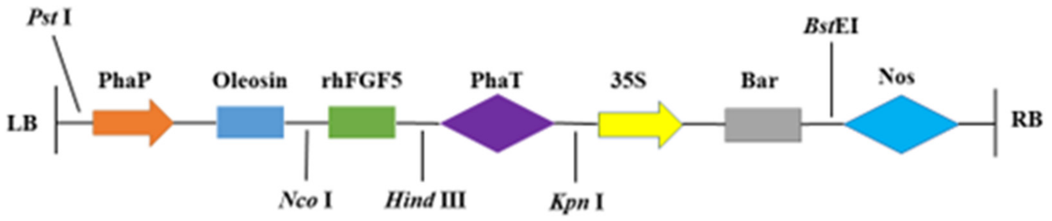

Figure S7. pOTB-hFGF5 expression vector. PhaP: phaseolin promoter; Oleosin: Arabidopsis thaliana oleosin gene; hFGF5: human fibroblast growth factor 5; PhaT: phaseolin terminator; 35S: CaMV35S promoter; Bar: the glufosinate resistance gene; Nos: Nopaline synthase terminator.

Table S1. Sequence of primers.

Primer	Sequence of primers 5'to3'
1-E2F5-F	GAAATGGGTCAGAATGGACAAAA
1-E2F5-R	AACCACGGGCTTAGATGAACTC
2-ID2-F	GAACAAGAAGGTGAGCAAGATGG
2-ID2-R	CTGACAATAGTGGGATGCGAGT
3-BAX-F	TCCCACCTTCCTAAATGTCTGTC
3-BAX-R	GCATCCACCGCACACTAAAGA
4-SMAD7-F	GGGAGGAGAAGACGAGAGTGG
4-SMAD7-R	CTTGTTGTCCGAATTGAGCTGT
5-E2F4-F	TGGAAGGTATCGGGCTAATCG
5-E2F4-R	ATGAGTGACGTAGGCCAAAGTGT
6-CSNK1E-F	TTCAACTTCTGTTCCCGCAAA
6-CSNK1E-R	GTGGATGAAGTTCTTGGAGTGGA
7-PIK3R3-F	CTCTTCCACCAAAGCCACCTAA
7-PIK3R3-R	TTGAAATATCCCCCCAGTACCA
8-SOS1-F	GGAACTATTCTTCTACCCCATGG
8-SOS1-R	CTCCACCTAAGGGCATCCAA
